# Catheter related blood stream infection (CRBSI) rate reduction program in a cardiac intensive care unit in Kualalumpur, Malaysia

**DOI:** 10.1017/ash.2025.112

**Published:** 2025-09-03

**Authors:** Vanitha Malar Nachiappan, Angelica Peter John

**Affiliations:** 1 Nurse Mentor, Adult Intensive Care Unit; 2 National Heart Institute, Malaysia; 3 Senior Staff Nurse, Adult Intensive Care Unit National Heart Institute, Malaysia

**Keywords:** Catheteter Related Blood Stream Infection, Central Line Maintenance Bundle

## Abstract

**Introduction:** Catheter Related Bloodstream Infection (CRBSI) continues to be a major healthcare associated infection in Intensive Care Units. The necessity of Central Venous Catheters (CVC) for critically ill patients creates a significant challenge in reducing the CRBSI rates. This challenge is further amplified by the extended duration of CVC use required for many of our patients due to complex treatment regimens, hemodynamic monitoring needs, and the presence of high comorbidity factors. The purpose of this study was to review the impact of a 12-month comprehensive CRBSI reduction program and a new Central Line Maintenance Bundle (CLMB) on reducing CRBSI rate in a cardiothoracic surgical ICU. **Case Presentation:** Observational study carried out and yearly CRBSI rate compared before and after implementation of the program. The program consists of one-month hand hygiene campaign conducted every quarterly throughout 2021, application of disinfectant cap (Curos) for all long staying patients, application of Chlorhexidine tegaderm for newly inserted central line, application of no sting barrier film at CVL sites and Adenosine triphosphate test on random surfaces for cleanliness. Besides, the existing CRBSI Bundle was separated into CRBSI Insertion and Maintenance Bundle. The new CRBSI Maintenance Bundle consists of HH, hub care, site care, tubing care and daily review. Infection control link nurses were appointed every shift to ensure adherence to the infection control protocol. The CRBSI rate before and after the program was compared. **Discussion:** At the beginning of the study, the CRBSI rate was 4.8 per 1000 catheter days. The CRBSI rate reduced to 3.3 per 1000 catheter days, at the end of the study. The overall reduction of CRBSI rate was 31%. Conclusion Implementing a comprehensive CRBSI reduction program and a new Central Line Maintenance Bundle was able to significantly reduce catheter related blood stream infection in ICU.

**Acknowledgments**: Department of Nursing, National Heart Institute, Dato Dr.Suneta Sulaiman, Intensive Care Unit Director.

